# Supplementary material for: Pareto optimality between growth-rate and lag-time couples metabolic noise to phenotypic heterogeneity in Escherichia coli
Source: Nat Commun. 2021 May 28;12:3204. doi: 10.1038/s41467-021-23522-0 (PMC8163773; doi:10.1038/s41467-021-23522-0)
Supplement: Supplementary file 1 — Supplementary Information [file 41467_2021_23522_MOESM1_ESM.docx]

**Supplementary Materials for**

**Pareto optimality between growth-rate and lag-time couples metabolic noise to phenotypic heterogeneity in Escherichia coli.**

Diego Antonio Fernandez Fuentes, Pablo Manfredi, Urs Jenal and Mattia Zampieri.

Correspondence to: zampieri@imsb.biol.ethz.ch

**This PDF file includes:**

Supplementary Text

Figs. S1 to S7

Supplementary Text

Lag time vs growth rate

Here we consider a simple exponential model of bacterial growth, in which the number of doublings after a period of time t (N(t)/N_0_, where N_0_ represents the initial number of cells) is a function of growth rate (ν) and lag time (t_lag_) – i.e. time it takes to the cell to start dividing. In a fixed period of time (t_max_), corresponding to θ number of cell duplications, growth rate in inversely proportional to lag time.

$$\begin{matrix} N(t)=N_{0} e^{\nu(t-t_{lag})} \\ \frac{\ln(N(t_{\max}))-\ln(N_{0})}{t_{\max}-t_{lag}}=\nu\\ \frac{\theta}{t_{\max}-t_{lag}}=\nu\end{matrix}$$

It has been demonstrated that in optimally growing *E. coli* cells, growth rate is proportional to the fermentative rate (r_f_) – i.e. acetate secretion rate, within a large range of growth rates ^1^. It is plausible to assume that in equally fit cells (isoclines in Fig4. A), carbon uptake is a constant (r_C_). Thereby, we expect colonies on the Pareto front to exhibit growth rate that negatively scale with respiratory rate (r_r_): $\nu\propto r_{f}=r_{C}-r_{r}$

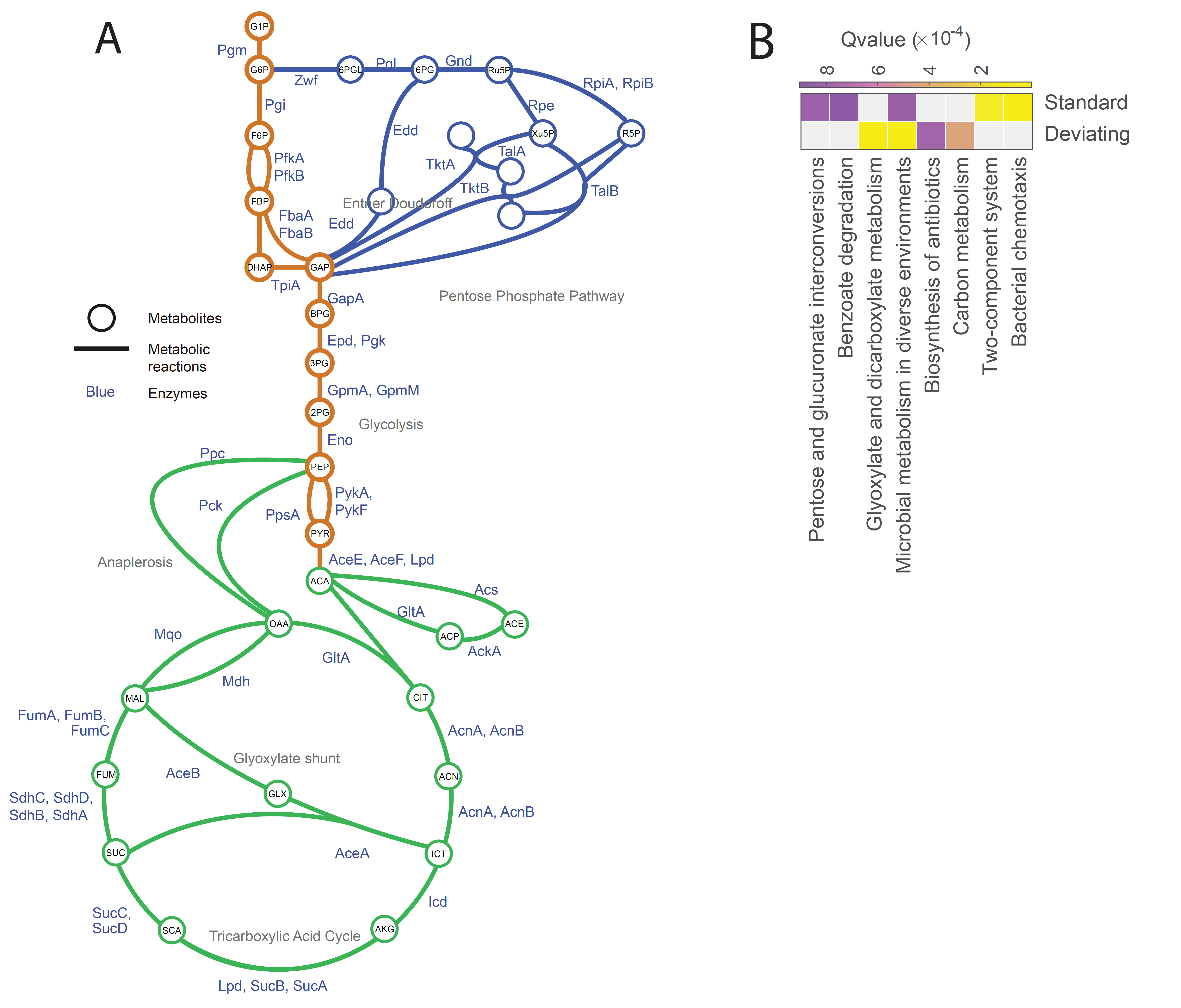

Fig. S1.

**Analysis of protein noise.** (A) Schematic representation of E. coli central metabolic network. Color coding of metabolic reactions are consistent with Figure 1C of the main text: Blue – pentose phosphate pathway, entner doudoroff pathway, Orange – upper and lower glycolysis, Green – tricarboxylic acid cycle, glyoxylate shunt and anaplerosis. (B) Enrichment of KEGG metabolic pathways for proteins with high standard and deviating noise. Only pathways with qvalue<0.001 are reported.


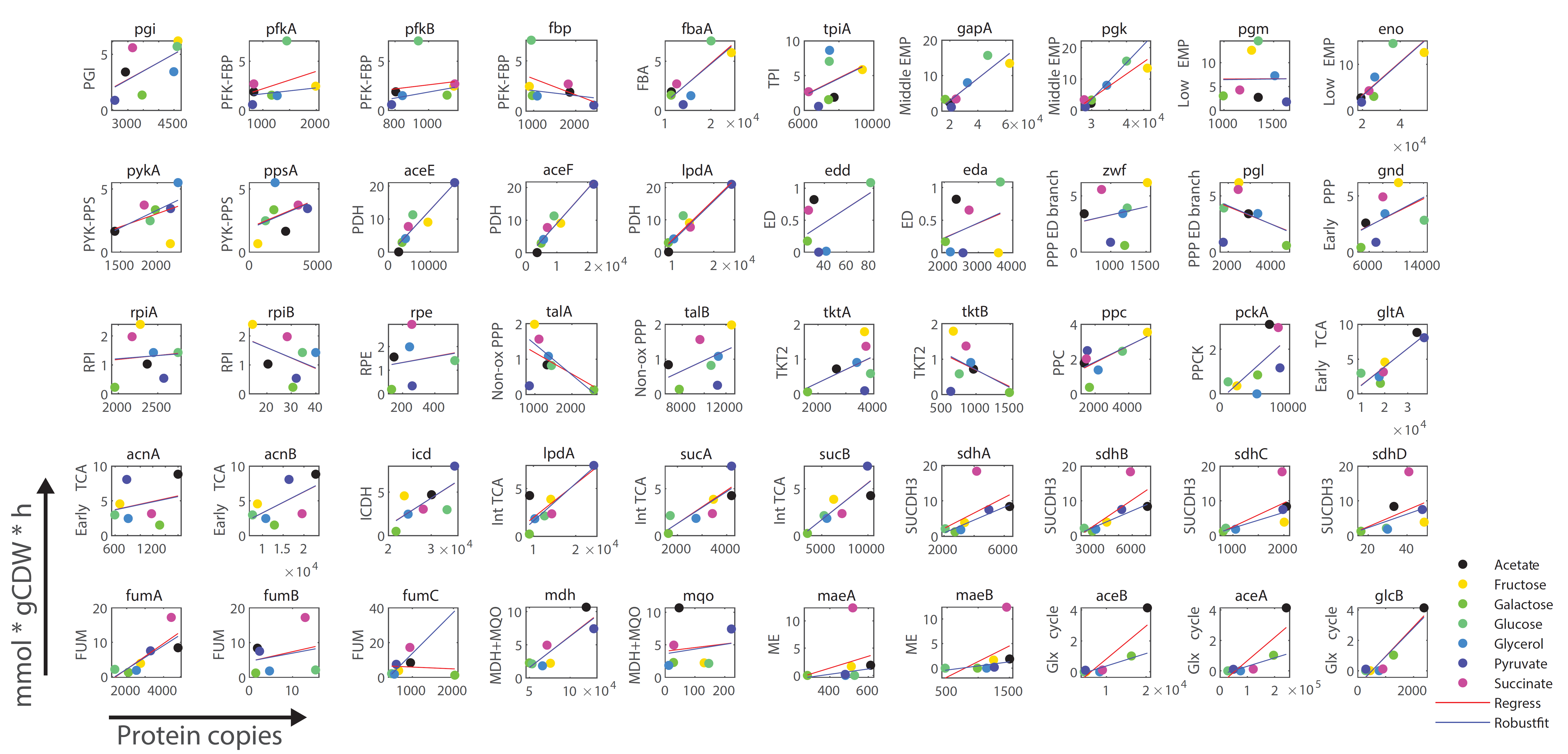


**Fig. S2.**

**Linear dependency between proteins and metabolic fluxes.** Linear least squares regression analysis of bulk measurements of protein copy numbers ^2^ and metabolic fluxes ^3^ in E. coli growing in minimal media with 7 different carbon sources. For each enzyme we find the best linear fit that explain changes in fluxes by protein copy numbers. We performed the analysis using ordinary least squares regression on all data points (red line). To test the sensitivity of our results to outliers in the data we also applied an iteratively reweighted least squares algorithm (blue line), with the weights at each iteration calculated by applying the bisquare function to the residuals from the previous iteration (robustfit function in Matlab). Results are largely consistent between the two methods.





**Fig. S3.**

**Monitoring colony growth.** (A) Schematics of monitoring colony growth trajectories and respiratory activity. Images of petri dishes are acquired every 10 minutes. First, we use an in-house software for colony segmentation and tracking, from which we derive position, size and average RGB color intensity for each colony (<http://www.imsb.ethz.ch/research/zampieri-group/resources.html>). (B) Next, individual colony trajectories are analyzed to estimate maximum growth-rate, lag-time and maximum respiratory-activity. (C) Changes of area over time for a wild-type *E. coli* colony growing in LB agar (blu dots). Best fit of a Gompertz growth function and 95% confidence intervals (red line and shaded region). The black cross highlights the time at which maximum growth-rate is achieved (t_m_). (D) Average red intensity measured for the same colony reported in panel c. Maximum respiratory rate is estimated by finding the time window consisting of at least 10 data points with the largest proportionality coefficient (α). (E-F) Distribution of lag-times and growth-rates for colonies in 3 separate dishes (i.e. biological replicates) growing in LB and LB+TCC agar plates. On average, TCC has the expected effect of reducing maximum growth rates and increasing lag-time, without inducing significant changes in colony-to-colony variability (i.e. CVs). (G) Distributions of times at which maximum growth rates (i.e. t_m_) (green) and respiratory rates (purple) are achieved by each colonies growing on LB+TCC agar plates of panel E and F. For the vast majority of colonies, maximum growth rate and respiratory activity are reached within few hours after detection of the colony.

**Fig. S4.**

**Experimentally testing the effects of cra overexpression/deletion on colony-to-colony variability.** Probability Density Function estimated from respiratory activity (A), lag time (B) and growth rate (C) of wild-type (blue), Δcra (red) and cra+ mutant (green) colonies growing in LB medium. For each parameter, the coefficients of variation (CV) are reported on the top of the panel.


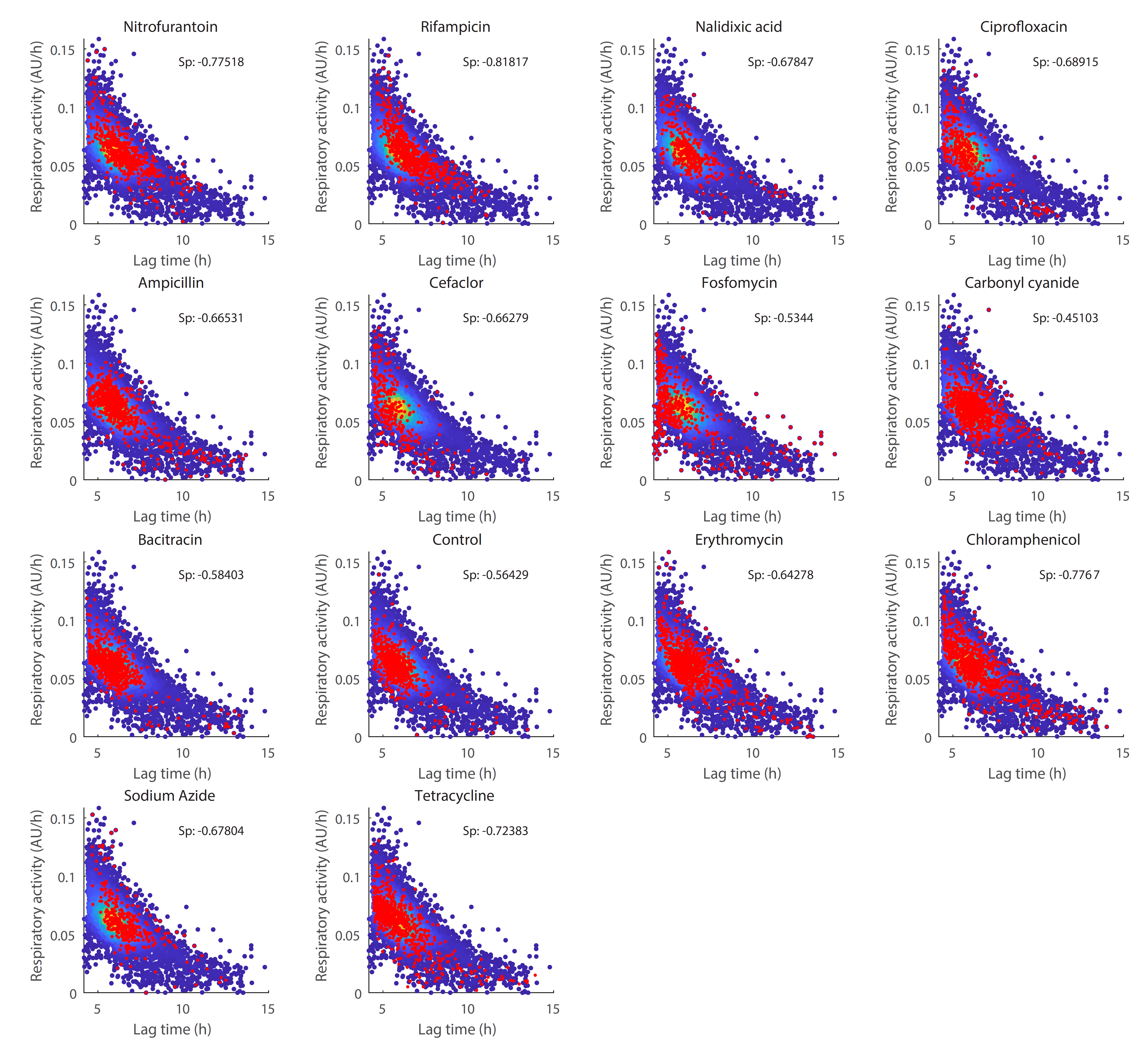


**Fig. S5.**

**Relationship between lag time and respiratory activity.** 2D density plots of estimates for individual colonies’ lag-time and respiratory activity in all 14 tested conditions (same as Figure 3 of the main text). In order to show that in all tested conditions/perturbations we found a strong anticorrelation between lag time and respiratory activity, colonies from each of the individual condition are highlighted in red and Spearman correlation (Sp) is reported on the upper-right corner of each panel.


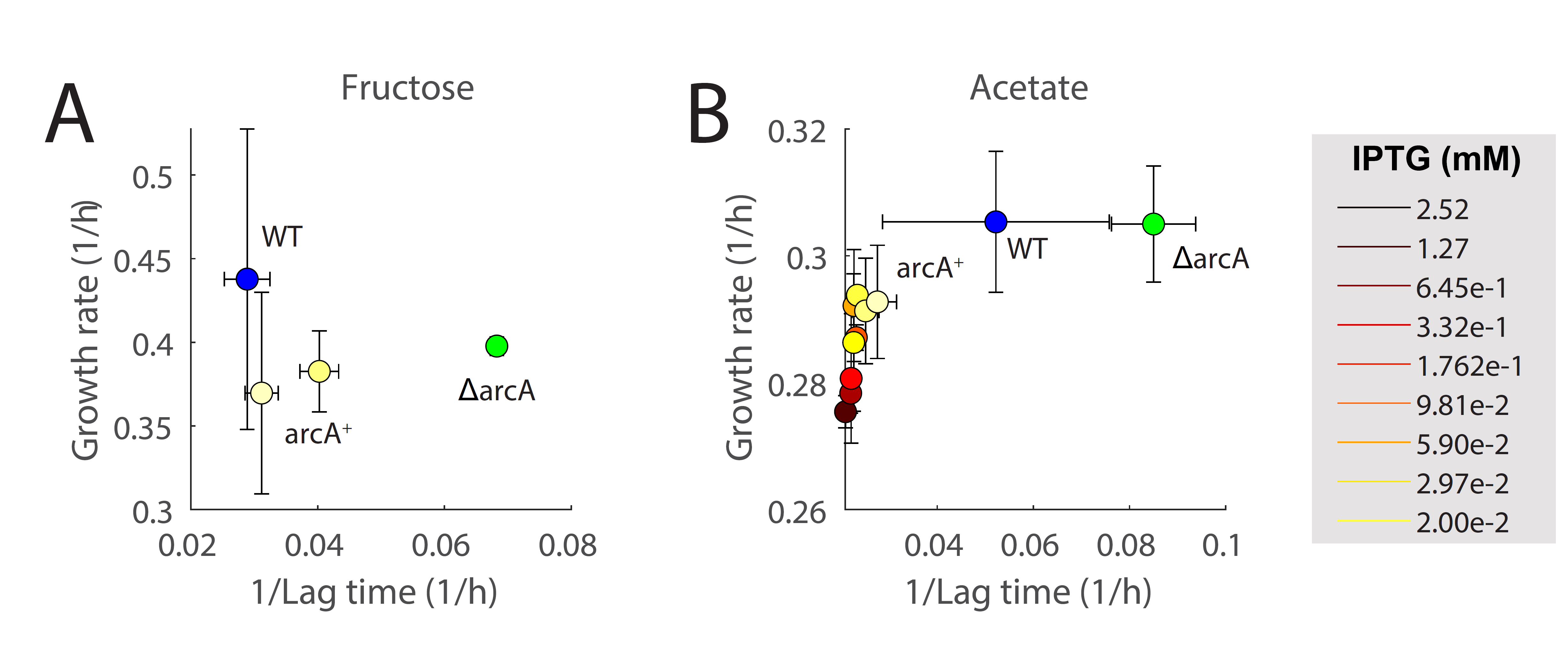


**Fig. S6.**

**Characterization of arcA overexpression**. Lag time vs growth rates in glucose minimal medium after 2 hours of carbon starvation in wild-type, ΔarcA and arcA^+^ mutants of E. coli. We grew wild-type E. coli, the ΔarcA and arcA^+^ mutants in batch cultures of glucose minimal medium up to mid-exponential phase (Optical Density at 600 nm - OD_600_ ~ 1), washed the cells and incubated them in M9 medium without carbon sources together with the IPTG inducer (0.02 mM) for 2 hours. Next, we resuspended cells in M9 medium with fructose (A) or acetate (B) and monitored OD_600_ in a plate reader using different levels of IPTG inducer. Reported data are average ± standard deviation over 3 biological replicates.


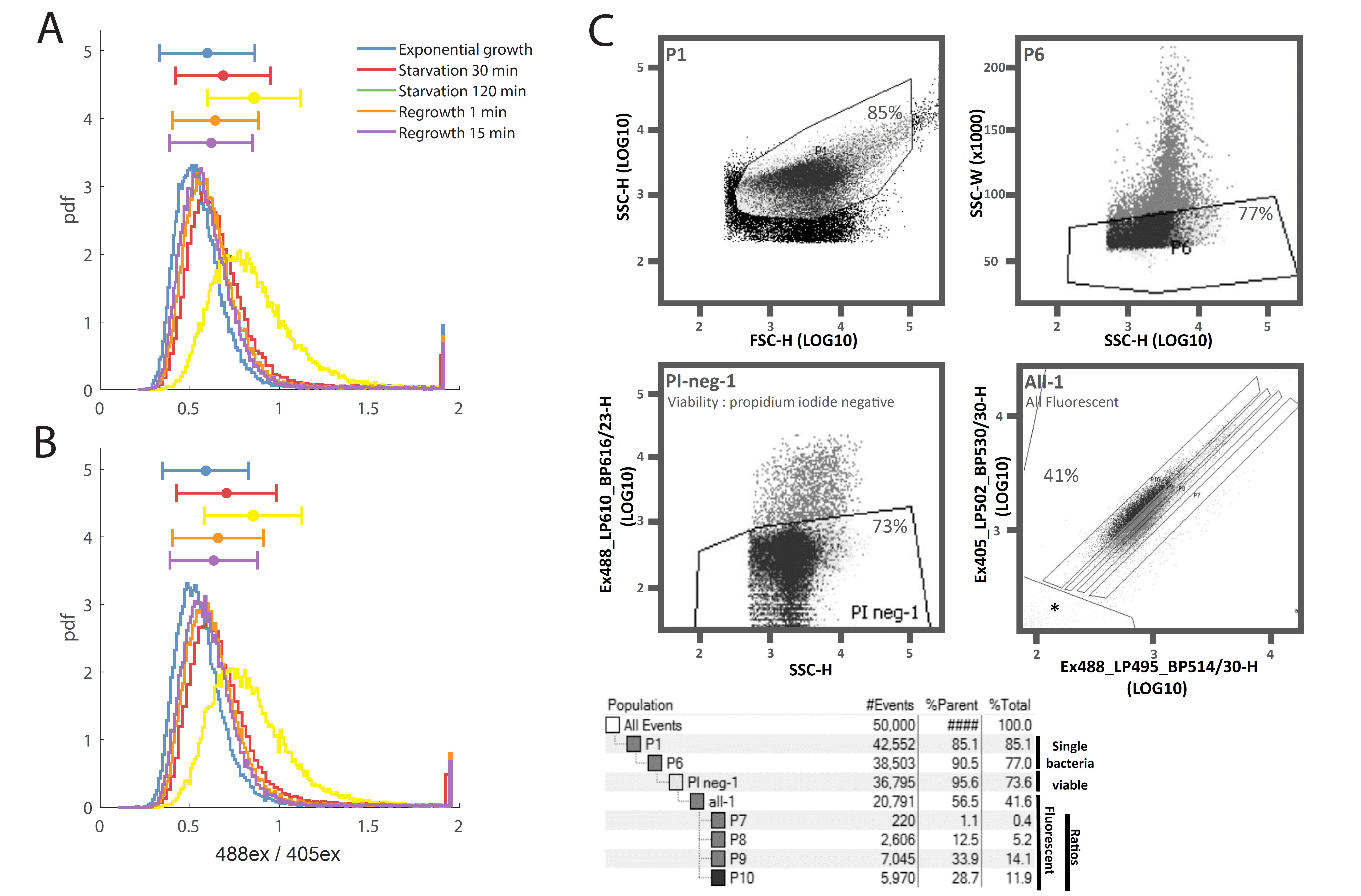


**Fig. S7.**

**Monitoring ATP in single cells of wild type and ΔarcA *E. coli***.

(A-B) We used fluorescence activated cell sorting (FACS) to measure depletion of ATP in single cells^4^ from wild type (panel A) and ΔarcA (panel B). Fluorescence was measured with the following channels Ex488_LP495_BP514/30-H, Ex405_LP502_BP530/30-H. The lower the ratio between 488 nm and 405 nm, the higher the ATP levels. We detect almost identical distribution (reported as probability density function -pdf) of ATP levels between exponentially growing populations of ΔarcA and wild type (blue), during carbon starvation (30 and 120 after suspending cells in M9 without carbon), 1 and 15 minutes after reintroducing glucose in the medium. (C) Gating strategy for detection of E. coli cells with different ATP concentration. FSC and SSC gatings for single bacteria capture. (P1 -> P6). Viable cells (PI-neg-1) were captured on a gate defined by an unstained sample. Fluorescent cells (All-1) were captured on a gate negatively defined by a strain expressing a non fluorescent variant of the ATP reporter (corresponding to the region marked with “*”). Percentage on the graphics refer to total population captured. P7, P8, P9 and P10 gates capture most of the fluorescent cells at different signals ratio between the Ex405_LP502_BP530/30-H and the Ex488_LP495_BP514/30-H channels.

**References**

1. Basan, M. *et al.* Overflow metabolism in Escherichia coli results from efficient proteome allocation. *Nature* **528**, 99–104 (2015).

2. Schmidt, A. *et al.* The quantitative and condition-dependent Escherichia coli proteome. *Nat. Biotechnol.* **34**, 104–110 (2016).

3. Gerosa, L. *et al.* Pseudo-transition Analysis Identifies the Key Regulators of Dynamic Metabolic Adaptations from Steady-State Data. *Cell Syst.* **1**, 270–282 (2015).

4. Yaginuma, H. *et al.* Diversity in ATP concentrations in a single bacterial cell population revealed by quantitative single-cell imaging. *Sci. Rep.* **4**, 1–7 (2014).
