# Supplementary material for: Pareto optimality between growth-rate and lag-time couples metabolic noise to phenotypic heterogeneity in Escherichia coli
Source: Nat Commun. 2021 May 28;12:3204. doi: 10.1038/s41467-021-23522-0 (PMC8163773; doi:10.1038/s41467-021-23522-0)
Supplement: Supplementary file 3 — Description of Additional Supplementary Files [file 41467_2021_23522_MOESM3_ESM.pdf]

Description of Additional Supplementary Files

Supplementary Data 1

Standard -Deviating noise: Standard and deviating protein noise levels estimated from data reported in (Taniguchi et al., 2010).

GO_enrichment: Gene ontology enrichment of proteins with largest standard or deviating noise levels. For each functional group, we reported the number of proteins with estimated noise levels, pvalues and pvalues corrected for multiple tests (i.e. qvalues).

Protein vs flux: We quantified the linear dependency between changes in 40 enzymes copy number (Schmidt et al., 2016) and 25 rates of the corresponding metabolic reactions (Gerosa et al., 2015) in E. coli growing in minimal media with 7 different carbon sources. For each protein, the coefficients (i.e. coefficient of proportionality and intercept) of the linear dependency between protein levels and fluxes are reported together with pvalues testing the null hypothesis that the variables are non correlated.

M9 / LB colonies: Maximum growth rate, lag time and respiratory activity colonies of wild type E. coli cells grown either in M9 glucose or LB agar plates.

Cra+ - Cra knockout: Maximum growth rate, lag time and respiratory activity for colonies of wild type, cra overexpression and knockout mutants of E. coli grown in LB agar plates.

Perturbations: List of perturbing agents. For each perturbing agent we reported the target cellular process (i.e. mode of action) and the amount spotted in the center of the petri dish.

Perturbation colonies: Maximum growth rate, lag time and respiratory activity for colonies of wild type, mutants grown in LB agar plates with perturbing agent applied at the center of the plate. For each colony the distance from the center is reported.

arcA vs wt SteadyState: For each annotated ion, metabolite name, KEGG id and mass-to-charge ratio are reported, together with averaged $\log_2$ fold change, standard deviation (across 3 biological replicates) and corrected pvalue (qvalue) estimated between exponentially growing wild-type and Δ*arcA* knockout mutants.

arcA vs wt 2hours starvation: For each annotated ion, metabolite name, KEGG id and mass-to-charge ratio are reported, together with averaged $\log_2$ fold change, standard deviation (across 3 biological replicates) and corrected pvalue (qvalue) estimated between wild-type and Δ*arcA* knockout mutants after 2 hours of carbon starvation.

WT dynamics starvation: For each annotated ion, metabolite name, KEGG id and mass-to-charge ratio are reported, together with averaged log2 fold change and standard deviation (across 3 biological replicates) estimated after 5, 10,15, 30, 60, 90, 120 minutes of starvation, with respect to metabolite abundances immediately after resuspending wild-type cells in M9 medium without carbon (i.e. time 0).

Δ*arcA* dynamics starvation: For each annotated ion, metabolite name, KEGG id and mass-to-charge ratio are reported, together with averaged log2 fold change and standard deviation (across 3 biological replicates) estimated after 5, 10,15, 30, 60, 90, 120 minutes of starvation, with respect to metabolite abundances immediately after resuspending Δ*arcA* cells in M9 medium without carbon (i.e. time 0).
