# Supplementary material for: Pareto optimality between growth-rate and lag-time couples metabolic noise to phenotypic heterogeneity in Escherichia coli
Source: Nat Commun. 2021 May 28;12:3204. doi: 10.1038/s41467-021-23522-0 (PMC8163773; doi:10.1038/s41467-021-23522-0)
Supplement: Supplementary file 5 — Reporting Summary [file 41467_2021_23522_MOESM5_ESM.pdf]

nature research

Corresponding author(s): Mattia Zampieri

Last updated by author(s): Jan 12, 2021

Reporting Summary

Nature Research wishes to improve the reproducibility of the work that we publish. This form provides structure for consistency and transparency in reporting. For further information on Nature Research policies, see our Editorial Policies and the Editorial Policy Checklist.

Statistics

For all statistical analyses, confirm that the following items are present in the figure legend, table legend, main text, or Methods section.

n/a	Confirmed	
☐	☒	The exact sample size (*n*) for each experimental group/condition, given as a discrete number and unit of measurement
☐	☒	A statement on whether measurements were taken from distinct samples or whether the same sample was measured repeatedly
☐	☒	The statistical test(s) used AND whether they are one- or two-sided<br>*Only common tests should be described solely by name; describe more complex techniques in the Methods section.*
☐	☒	A description of all covariates tested
☐	☒	A description of any assumptions or corrections, such as tests of normality and adjustment for multiple comparisons
☐	☒	A full description of the statistical parameters including central tendency (e.g. means) or other basic estimates (e.g. regression coefficient) AND variation (e.g. standard deviation) or associated estimates of uncertainty (e.g. confidence intervals)
☐	☒	For null hypothesis testing, the test statistic (e.g. *F*, *t*, *r*) with confidence intervals, effect sizes, degrees of freedom and *P* value noted<br>*Give P values as exact values whenever suitable.*
☒	☐	For Bayesian analysis, information on the choice of priors and Markov chain Monte Carlo settings
☐	☒	For hierarchical and complex designs, identification of the appropriate level for tests and full reporting of outcomes
☐	☒	Estimates of effect sizes (e.g. Cohen's *d*, Pearson's *r*), indicating how they were calculated

Our web collection on statistics for biologists contains articles on many of the points above.

Software and code

Policy information about availability of computer code

Data collection	All code used to generate the data used in the manuscript is specified in the methods section or made it available publicly through the GitHub API (https://github.com/Dfernand1795/PetriScanner2). The software is based on Python and requires ImageJ - Fiji version 2.0
Data analysis	All code used to analyze the data used in the manuscript is specified in the methods section or made it available publicly through the GitHub API (https://github.com/Dfernand1795/PetriScanner2). Visualization of data reported as Supplementary materials were generated in Matlab R2018 (MathWorks).

For manuscripts utilizing custom algorithms or software that are central to the research but not yet described in published literature, software must be made available to editors and reviewers. We strongly encourage code deposition in a community repository (e.g. GitHub). See the Nature Research guidelines for submitting code & software for further information.

Data

Policy information about availability of data

All manuscripts must include a data availability statement. This statement should provide the following information, where applicable:

- Accession codes, unique identifiers, or web links for publicly available datasets
- A list of figures that have associated raw data
- A description of any restrictions on data availability

All data generated or analyzed during this study are included in this published article as Supplementary Data. A detailed description of all data analysis steps is published in this article in the Methods section and Supplementary Information. Python code for the image analysis software is available for download at http://www.imsb.ethz.ch/research/zampieri-group/resources.html.

Field-specific reporting

Please select the one below that is the best fit for your research. If you are not sure, read the appropriate sections before making your selection.

- [x] Life sciences
- [ ] Behavioural & social sciences
- [ ] Ecological, evolutionary & environmental sciences

For a reference copy of the document with all sections, see nature.com/documents/nr-reporting-summary-flat.pdf

Life sciences study design

All studies must disclose on these points even when the disclosure is negative.

Sample size	For experiments involving quantification of colony growth characteristics and bulk measurements of growth, lag time and metabolome in liquid cultures, n≥3 was chosen as the replicate number, and sample size was determined by reproducibility analysis and low observed variability between replicates.
Data exclusions	No data were excluded from analysis
Replication	Metabolome measurements and estimations of growth rates and lag time were performed in triplicates to estimate errors associated with optical density and mass spectrometry measurements. All attempts of replication were successful.
Randomization	Randomization was used to determine differences in colony-to-colony variability between different mutant strains of E. coli (Fig. 2 panels F-G-H). Here we used the fucntion "randperm" in Matlab R2018 and estimated the statistics from 1000 random selections of 400 colonies from each mutant.
Blinding	Blinding was not adopted as the conditions were well characterized. Quantification of differences between conditions were performed using computational analysis applied equally to all conditions and replicates.

Reporting for specific materials, systems and methods

We require information from authors about some types of materials, experimental systems and methods used in many studies. Here, indicate whether each material, system or method listed is relevant to your study. If you are not sure if a list item applies to your research, read the appropriate section before selecting a response.

Materials & experimental systems

n/a	Involved in the study	
x		Antibodies
x		Eukaryotic cell lines
x		Palaeontology and archaeology
x		Animals and other organisms
x		Human research participants
x		Clinical data
x		Dual use research of concern

Methods

n/a	Involved in the study	
x		ChIP-seq
	x	Flow cytometry
x		MRI-based neuroimaging

Flow Cytometry

Plots

Confirm that:

- [x] The axis labels state the marker and fluorochrome used (e.g. CD4-FITC).
- [x] The axis scales are clearly visible. Include numbers along axes only for bottom left plot of group (a 'group' is an analysis of identical markers).
- [x] All plots are contour plots with outliers or pseudocolor plots.
- [x] A numerical value for number of cells or percentage (with statistics) is provided.

Methodology

Sample preparation	The low concentration ATP reporter plasmid pRS-QUE7mu 21 was transformed into E.coli bacterial strains BW25113 and BW25113-ΔarcA (Keio:JW4364) expressing a T7 RNA polymerase (araB::T7RNAP-tetA). For each sample a single bacterial colony was grown in LB medium for a 6 hours at 37°C under agitation (170 RPM). 30 microliters of culture were pelleted, resuspended in 3 ml of M9 medium and further diluted 1:10 in 3 ml of M9 medium supplemented with Glucose

0.5%, ampicillin 50 µg/ml and arabinose 0.01%. Cultures were grown for 16 hours at 37°C under agitation (170 RPM) and typically reached OD600 values between 0.05-0.3. For the starvation procedure, cells were washed twice and resuspended in 3 mL of M9 medium only supplemented with ampicillin 50 µg/ml. Samples were brought back to the shaker for two hours before addition of glucose at 0.5%. Cultures were sampled for FACS analysis right before the starvation phase, after 30 and 120 minutes of starvation and after one and 15 minutes after addition of glucose. 30 µl of sampled culture was diluted in PBS containing propidium iodide (1µg/ml, ThermoFisher:P3566). FACS measurements were performed on a BD FACSaria III Cell sorter. Fluorescence was measured with the following channels Ex488_LP495_BP514/30-H, Ex405_LP502_BP530/30-H and Ex488_LP610_BP616/23-H (for viability; propidium iodide).

Instrument	BD FACSaria III Cell sorter
Software	Matlab
Cell population abundance	~10000
Gating strategy	Gating strategy for detection of E. coli cells with different ATP concentration. FSC and SSC gatings for single bacteria capture. (P1 -> P6). Viable cells (PI-neg-1) were captured on a gate defined by an unstained sample. Fluorescent cells (All-1) were captured on a gate negatively defined by a strain expressing a non fluorescent variant of the ATP reporter (corresponding to the region marked with "*"). Percentage on the graphics refer to total population captured. P7, P8, P9 and P10 gates capture most of the fluorescent cells at different signals ratio between the Ex405_LP502_BP530/30-H and the Ex488_LP495_BP514/30-H channels (Figure S7).

☒ Tick this box to confirm that a figure exemplifying the gating strategy is provided in the Supplementary Information.
